# Use of traditional and complementary medicine for musculoskeletal diseases

**DOI:** 10.3906/sag-1509-71

**Published:** 2019-06-18

**Authors:** Gülis KAVADAR, Saliha EROĞLU DEMİR, Ebru AYTEKİN, Yıldız AKBAL

**Affiliations:** 1 Department of Physical Medicine and Rehabilitation, Bahçeşehir Liv Hospital, İstinye University, İstanbul Turkey; 2 Department of Physical Medicine and Rehabilitation, Rumeli Hospital, İstanbul Turkey; 3 Department of Physical Medicine and Rehabilitation, İstanbul Education and Research Hospital, Health Sciences University,İstanbul Turkey; 4 Department of Physical Medicine and Rehabilitation, Faculty of Medicine, Bezmiâlem Vakıf University, İstanbul Turkey

**Keywords:** Complementary medicine, traditional medicine, musculoskeletal diseases

## Abstract

**Background/aim:**

We aimed to determine the prevalence of traditional and complementary medicine (TCM) use among patients with musculoskeletal disorders (MSDs) and to examine the methods used, frequency, and experienced benefits of therapies; the reasons for using TCM; and the sources of information about therapies. We also compared demographic characteristics of users and nonusers of TCM.

**Materials and methods:**

The descriptive study was conducted with 839 patients who attended the physical therapy and rehabilitation units of 3 public hospitals between September 2014 and March 2015. A self-administered questionnaire including demographic characteristics, medical history, and details of TCM use was applied.

**Results:**

Of the 839 individuals in the study (592 female, 247 male; mean age 48.9 ± 13.0 years), 35.4% reported using TCM. There was no significant statistical difference between users and nonusers in terms of age, gender, body mass index, socioeconomic status, or educational level (P > 0.05). The disease duration of TCM users was significantly higher than that of nonusers (P < 0.05). The most commonly used TCM methods were balneotherapy (31%), herbal therapies (30%), wet cupping (22.2%), and massage-manipulation methods (21.2%). Of TCM users, 75.1% were satisfied.

**Conclusion:**

The prevalence of TCM use and satisfaction levels are high in patients with MSDs. Physicians should be well informed about TCM methods and raise the awareness of patients to prevent improper use of TCM.

## 1. Introduction

Traditional and complementary medicine (TCM) can be defined as a group of diverse medical and healthcare systems, practices, or products that are designed to prevent, treat, or manage illnesses and preserve the mental and physical well-being of individuals (1). TCM methods can be classified into 5 major categories of practice: whole medical systems, mind-body techniques, biologically based therapies, manipulative and body-based therapies, and energy therapies (2). 

Although the clinical efficacy of many TCM methods is controversial because of a lack of scientific evidence, TCM use is increasing significantly throughout the world (3). In previous studies, chronic musculoskeletal pain caused by musculoskeletal disorders (MSDs), a common cause of chronic pain and physical disability, is one of the most reported conditions leading to TCM use (4–7). Medical, psychological, social, and economic costs of conventional treatments for MSDs and traditional or religious beliefs may be leading patients to seek different treatment methods (8,9). Understanding how and why patients choose TCM methods is important for chronic disease management.

There are few studies about TCM practices in Turkey and such studies have generally been conducted with cancer patients or among the general society, not patients with MSDs (10–12). The aims of this study were to determine the prevalence of TCM use among patients with MSDs and to examine the methods used, frequency, and experienced benefits of therapies; the reasons for using TCM; and the sources of information about therapies. We also compared the demographic characteristics of users and nonusers of TCM.

## 2. Materials and methods

This cross-sectional descriptive study was carried out from September 2014 to March 2015 among MSD patients who visited the physical therapy and rehabilitation units of 3 public hospitals. After a brief explanation of the purpose of the study, 839 volunteers between 18 and 86 years old who were able to understand all questions were included in the study. The study was approved by the local ethics committee. All individuals gave informed consent. The participants were interviewed face-to-face by means of a structured, self-administered questionnaire, which was created as a result of a review of the related literature (13–15).

The first part of the questionnaire was about sociodemographic characteristics: age, gender, body mass index (BMI), educational level, and income status. The second part was about medical characteristics: the nature and duration of the complaint and any previous treatments, including TCM use. The third part was for patients who had used any kind of TCM for musculoskeletal problems at some point in their lives. Participants were asked about their current or previous use of the following categories of TCM for MSDs: 1) whole medical systems (acupuncture, homeopathy, and naturopathy), 2) mind and body practices (meditation, hypnotherapy, and relaxation techniques), 3) biologically based supplements and topical agents (herbs, vitamins, minerals, and nutritional supplements), 4) manipulative and body-based practices (massage, cupping therapy [dry or wet], hirudotherapy, chiropractic or osteopathic manipulation, balneotherapy, reflexology, and neural therapy), and 5) energy medicine (healing, reiki, and magnetic therapy) (http://nccam.nih.gov/health/whatiscam).

Questions in the third section about the reasons for using TCM included any source of recommendation (relatives, media, or healthcare practitioners), the practitioner of the methods (educated, not educated, or self-educated), satisfaction from the methods (yes, no, or not sure), whether the patient considered using TCM again in the future (yes, no, or not sure), and if the patient would recommend TCM to someone else (yes, no, or not sure).

### 2.1. Statistical analysis

Statistical analysis was performed using SPSS 20.0 for Windows (IBM Corp., Armonk, NY, USA). Descriptive data were presented as mean ± standard deviation (SD). Categorical data were given as counts (n) and percentages (%). The conformability of the data to normal distribution was tested with the Kolmogorov–Smirnov test. Comparison of categorical variables was carried out using Pearson’s chi-square test. Normally distributed parameters were evaluated with the Student t-test, whereas variables without a normal distribution were assessed by the Mann–Whitney U test between groups. The confidence interval was set at 95% and the level of statistical significance was set at P < 0.05.

## 3. Results

A total of 839 patients (592 female, 247 male) were interviewed in the study. The distribution of demographic and clinical characteristics of the patients is summarized in Table 1.

**Table 1:** Distribution of demographic and clinical characteristics of patients.

TCM		Nonuser(n: 542)	User(n: 297)
Gender, female/male, n		376/166	216/81
Age, years, mean ± SD		48.9 ± 13.3	49.0 ± 12.5
BMI, kg/m2		27.8 ± 5.0	28.3 ± 5.1
Disease duration, years		3.3 ± 4.8	5.3 ± 6.4
Income status,n (%)	Low (0–1500 Turkish lira/month)	158 (29.2)	79 (26.6)	Middle (1500–3000 Turkish lira/month)	313 (57.7)	173 (58.2)	High (>3000 Turkish lira /month)	46 (8.5)	27 (9.1)	No answer	25 (4.6)	18 (6.1)
	Edu., n (%)	None	71 (13.1)	37 (12.5)
		Elementary	258 (47.6)	126 (42.4)
		Secondary	65 (12)	40 (13.5)
	High school	93 (17.2)	56 (18.9)
	University	54 (10)	35 (11.8)
	No answer	1 (0.2)	3 (1)

BMI: Body mass index, Edu.: education level.

At least one type of TCM for MSDs was reported by 297 (35.4%) of patients. The mean ages were 48.9 ± 13.3 and 49.0 ± 12.5 years for nonusers and users, respectively. There was no significant statistical difference between the two groups in terms of age, gender, BMI, income status, or educational level (Table 1, P > 0.05). The disease duration was 3.3 ± 4.8 and 5.3 ± 6.4 years in the nonuser and user groups, respectively (Table 1, P < 0.05).

Disease types of users and nonusers are shown in Table 2.

**Table 2:** Disease types of users and nonusers of TCM.

	Nonuser		User
	n	%	n
Intervertebral disc diseases	181	56	96
Spondylosis/osteoarthritis	112	20.7	88
Connective tissue problems	164	65.9	85
Neurologic (entrapment syndromes, neuropathy, etc.)	32	91.4	3
Inflammatory joint diseases	27	80	18
Metabolic bone diseases	13	86.7	2
Fracture, skeletal deformities	13	72.2	5

Sources of recommendations, statements about satisfaction, willingness to receive again, and willingness to suggest the method to someone else are shown for each TCM method in Table 3. The most-used TCM methods were balneotherapy (31%), herbal therapies (30%), wet cupping (bloodletting) (22.2%), massage-manipulation methods (21.2%), dry cupping (12.8%), hirudotherapy (8.1%), and acupuncture (4%). The satisfaction rates were 74% for balneotherapy, 59% for herbal therapies, 63% for bloodletting, 79% for massage-manipulation methods, 89% for dry cupping, 68% for hirudotherapy, and 85% for acupuncture. The percentage of patients considering receiving the method again for balneotherapy, herbal therapy, wet cupping, massage-manipulation, dry cupping, hirudotherapy, and acupuncture was 82%, 56%, 66%, 71%, 81%, 68%, and 71%, respectively. The percentage of patients considering suggesting the method to someone else for balneotherapy, herbal therapy, wet cupping, massage-manipulation, dry cupping, hirudotherapy, and acupuncture was 81%, 54%, 71%, 73%, 81%, 72%, and 78%, respectively. Most of the methods were recommended by relatives. The most recommended methods by healthcare practitioners were herbal therapy, balneotherapy, and acupuncture (Table 3).

**Table 3:** Characteristics of TCM users.

	Source of recommendation	Benefit	Repeat again	Recommend
	HCP/media/relatives	Y/N/NS	Y/N/NS	Y/N/NS
Balneotherapy (93)	18/0/75	69/20/4	77/12/4	76/12/5
Herbal treatment (92)	26/23/43	55/26/11	52/28/12	50/26/16
Wet cupping (66)	0/2/64	42/19/5	44/17/5	47/16/3
Massage-manipulation (63)	4/0/59	50/11/2	45/16/2	46/13/4
Dry cupping (38)	1/2/35	34/4/0	31/6/1	31/6/1
Hirudotherapy (25)	0/1/24	17/6/2	17/6/2	18/5/2
Acupuncture (14)	9/0/5	12/1/1	10/2/2	11/0/3
Other (12)	4/0/8	7/2/3	6/2/4	6/2/4

HCP: Healthcare practitioner, Y: yes, N: no, NS: not sure.

Of TCM users, 73.4% had tried only one method and 21.9% had used two methods (Table 4). The percentage of TCM users listing dissatisfaction with conventional treatments as the reason for trying TCM was 17.4% for herbal therapy, 34.9% for massage-manipulation, 57% for balneotherapy, 71.4% for acupuncture, 56.1% for wet cupping, 48% for hirudotherapy, 50% for dry cupping, and 66.7% for other TCM methods.

**Table 4:** Number of TCM methods used.

Number of TCM methods used	N	%
1	218	73.4
2	65	21.9
3	9	3.0
4	4	1.3
5	1	0.3

## 4. Discussion

In this study, the prevalence of TCM use for MSDs was 35.4% and 75.1% of TCM users were satisfied with the methods used. Epidemiological data indicate that TCM use for MSDs in developed countries ranges between 20% and 80% (16). In studies performed in Turkey, Ulusoy et al. found that 46.2% of patients with rheumatic diseases had used TCM methods; in the study of Mollaoğlu et al., the prevalence of TCM use in chronic diseases was 55.9%, and in the study of Tokem et al., 46.9% of patients with rheumatoid arthritis reported the use of TCM therapies (7,17,18). Social, cultural, and economic factors, as well as traditional structures of societies, may affect TCM use.

In our study, 73% of patients using TCM were females, 42.4% were elementary school graduates, and 58.2% were of middle income status. The study group consisted mostly of these patients. Patients with a longer disease duration were most likely to use TCM. In a study involving 5750 patients with chronic pain, education, pain severity, and pain duration were found to be the persistent correlates of TCM usage (19).

In this study, balneotherapy, herbal therapies, bloodletting (wet cupping), massage-manipulation methods, dry cupping, and hirudotherapy were the most-used TCM methods.

Balneotherapy has been a popular treatment modality for centuries in many countries that uses the physical and chemical effects of water for well-being. In the study of Umay et al., balneotherapy was found to be more effective than standard physical therapy for MSDs in terms of pain, satisfaction status, and analgesic drug consumption (20). Of the TCM users in our study, 31% had used balneotherapy for MSDs and 74% of them were satisfied with the treatment.

Herbal remedies, including the use of nutritional supplements and vitamins, is among the most popular TCM methods in Turkey and throughout the world. In terms of herbal variety, Turkey is one of the richest countries in the world and herbal therapy is a part of cultural tradition in Turkey. These remedies are considered natural and safe; however, many well-designed clinical and observational studies have identified many serious side effects like end-stage renal failure, liver damage, or neuropathy. In addition, if people use vitamins without a confirmed vitamin deficiency, they are exposed to the potential prooxidant effects of these synthetic products rather than their antioxidant characteristics (21–23). In our study, 59% of users reported satisfaction from herbal methods and no systemic side effects were detected. The most-used herbal remedies in our study were various herbs (used topically or orally) and nutritional supplements (glucosamine, chondroitin, hyaluronic acid, or collagen preparations). In this study, herbal remedies seem to be the TCM method recommended most often by medical staff, but only nutritional supplements, not herbs, were recommended by physicians. In recent years nutritional supplements have begun to be listed as nonpharmacological conventional treatments in some guidelines.

Another TCM method, cupping therapy, is an ancient medical treatment with two types, wet or dry. Wet cupping (bloodletting) relies on creating a local suction to mobilize blood flow from the painful area. The method is mostly used in Islamic countries and was found to be effective in some studies, but the method has a high risk of causing infection and bleeding (24). Unfortunately, 68.2% of wet cupping was applied by uneducated practitioners in our study. However, 63% of users were satisfied, 66% were considering receiving the treatment again, and 71% were considering proposing the method to someone else. Dry cupping was also a frequently used TCM method that was usually recommended and applied by relatives; 89% of patients were satisfied.

Hirudotherapy (leech therapy) is another popular method for MSDs. When applied inappropriately, serious side effects can be seen, ranging from allergic reactions to excess bleeding (25). Of our patients, 8.1% reported using leeches; 68% were applied by uneducated practitioners or the patient while 12% were applied by healthcare practitioners, and 68% of individuals were satisfied with the method.

Massage and manipulation methods for MSDs are licensed TCM therapies in many countries. A study in the United States found that back pain was the most common reason for visiting chiropractors and massage therapists (26). If applied properly, the methods are very effective in pain relief and increasing the range of motion. In our study 76% of individuals were satisfied; 50% had chosen uneducated practitioners.

We found that patients were not very interested in certain methods like acupuncture or neural therapy. The knowledge about these methods might be inadequate, and the implementation techniques and high costs may also affect their usage. In this study, only 4.7% of patients used acupuncture for MSDs; however, 85% of them were satisfied.

Dissatisfaction with conventional medical treatments was given as the reason for using TCM at rates of 17.4% for herbal therapy, 34.9% for massage-manipulation, 57% for balneotherapy, 71.4% for acupuncture, 56.1% for wet cupping, 48% for hirudotherapy, 50% for dry cupping, and 66.7% for other TCM users. In a study on patients attending clinics of rheumatology and orthopedics, 63% had already used or thought of using TCM even though they were satisfied with conventional treatments. In the same study, female patients and those who expressed dissatisfaction with conventional therapies were more likely to use TCM (27). In another study, 67% of patients with chronic pain were using TCM in conjunction with conventional methods (28). Unfortunately, clinicians are generally not aware of the use of TCM among their patients (27). The majority of TCM users find these healthcare alternatives safer and more personal. TCM methods may give rise to serious harm; however, the positive impacts on patients cannot be ignored. Therefore, physicians should take a detailed medical history, including TCM use, and must be aware of the physical, psychosocial, and spiritual needs of patients.

This study has some strengths and limitations. Notable strengths are evaluation of a relatively wide patient population from 3 different centers and assessment of the patients with MSDs only. One of the limitations of the study was that the data were collected according to patient reports. The second limitation was that participation in the study was on a voluntary basis; thus, a large portion of the voluntary participants may have been more involved with TCM use.

In conclusion, this study showed that TCM use is common among patients with MSDs and that their satisfaction levels are high. The TCM methods are usually applied by uneducated practitioners and the patients usually obtain information from their relatives. Therefore, physicians should be well informed about TCM methods and should raise the awareness levels of patients to prevent improper use of TCM. Further research is necessary about the efficacy, benefits, and risks of TCM methods for better insight.
